# Ensuring Quality Standards and Reproducible Research for Data Analysis Services in Oncology: A Cooperative Service Model

**DOI:** 10.3389/fcell.2019.00349

**Published:** 2019-12-17

**Authors:** Frank Emmert-Streib, Matthias Dehmer, Olli Yli-Harja

**Affiliations:** ^1^Predictive Society and Data Analytics Lab, Faculty of Information Technology and Communication Sciences, Tampere University, Tampere, Finland; ^2^Institute of Biosciences and Medical Technology, Tampere, Finland; ^3^Steyr School of Management, University of Applied Sciences Upper Austria, Steyr, Austria; ^4^Department of Mechatronics and Biomedical Computer Science, UMIT, Hall in Tyrol, Austria; ^5^College of Artificial Intelligence, Nankai University, Tianjin, China; ^6^Institute for Systems Biology, Seattle, WA, United States

**Keywords:** computational biology, biostatistics, genomics, reproducible research, oncology, precision medicine, data science

## Abstract

Modern molecular high-throughput devices, e.g., next-generation sequencing, have transformed medical research. Resulting data sets are usually high-dimensional on a genomic-scale providing multi-factorial information from intertwined molecular and cellular activities of genes and their products. This genomics-revolution installed precision medicine offering breathtaking opportunities for patient's diagnosis and treatment. However, due to the speed of these developments the quality standards of the involved data analyses are lacking behind, as exemplified by the infamous Duke Saga. In this paper, we argue in favor of a two-stage cooperative serve model that couples data generation and data analysis in the most beneficial way from the perspective of a patient to ensure data analysis quality standards including reproducible research.

## 1. Introduction

Every new era provides opportunities but also challenges. For instance, at the early stage of the Industrial Revolution several severe accidents happened, one of which was the explosion of a steam boiler at a brewery in Mannheim in 1865. This and similar incidents resulted in the establishment of the TÜV (english meaning is Technical Inspection Association) in Germany as an independent institution for providing inspection and product certification services. Since then from a high-tech nuclear power plant to a simple hair-dryer every factory, product or service needs to pass a mandatory safety test from this independent association before it can be put into operation. Different countries may have different implementation rules and enforcing institutions but essentially every western country follows this model for all produces and services. However, there is one notable exception to the above and this relates to the analysis services of biomedical data.

An unfortunate example that demonstrates the catastrophic consequences of the lack of quality control standards in data analysis services in genomic medicine is the Duke Saga (Kolata, 2011). Specifically, a re-analysis of cancer genomic studies conducted at the Duke University by Anil Potti by external experts (Keith Baggerly and Kevin Coombes) from the MD Anderson found that various publications contained fundamental flaws and even scientific misconduct (Baggerly and Coombes, 2009; Potti et al., 2011). These issues were so severe leading ultimately in the discontinuation of three clinical cancer trials that were started as a consequence of Anil Potti's research findings and the retraction of over ten scientific papers, all published in renowned journals, including the New England Journal of Medicine, Lancet Oncology and Nature Medicine. Sadly, further examples along these lines are ample (Ioannidis, 2005; Godlee et al., 2011; Simmons et al., 2011; Gupta, 2013; Tripathi et al., 2013).

## 2. Measures for Ensuring Quality Standards

In our opinion the Duke Saga has similarities to the steam boiler explosion in Mannheim and we need to draw similar consequences from this incident. Specifically, we suggest that data analysis processes applied to medical, clinical and biomedical data, from which conclusions are drawn that will be used for the diagnosis or treatment of patients, need to be approved and certified by an external association in order to minimize the risk for patients. Here two important parameters of such an external association are:

(A) The independence of the external association from the data generating institution.
(B) The demonstrated expertise of the members constituting the external association.

### 2.1. Independence of the External Association From the Data Generating Institution

With respect to point (A), the independence of the external association from the data supplying institution needs to include its financial independence. This is in fact a problem with the current system. Specifically, nearly every medical or clinical institution maintains nowadays departments for biostatistics or computational biology. However, the scientists employed in these departments can hardly make decisions that are not aligned to the strategic interests of the institution. In contrast, an external association that is financially independent doesn't have to consider such strategic directions, in fact, it must not consider these because flawed decisions can endanger the well-being of patients.

### 2.2. Demonstrated Expertise of the Members Constituting the External Association

With respect to point (B), it is important that the members of the external association have a PI status. This ensures the highest possible standards of the quality control service that is needed in a clinical context. This is necessary because the fast paced developments in cancer genomics and its data analysis processes require constantly novel solutions that are not available off-the-shelf (Dunn and Bourne, 2017; Emmert-Streib and Dehmer, 2019).

This is also in contrast to most biostatistics and computational biology departments at medical or clinical institutions, which serve frequently merely as facilities to provide support for other departments at the same institution. This implies also that such facilities usually don't have a budget for developing new methods or for finding these, e.g., by a comparative analysis.

### 2.3. Similarities to the FDA

A related organization that has some similarities to our envisioned external association is the Food and Drug Administration (FDA) of the United States Department of Health and Human Services. For instance, the FDA is involved in the approval of medications, vaccinations and cosmetics. However, in contrast to our model described above, the FDA is predominantly concerned about outcome rather than the process leading to an outcome. That means the FDA does not perform experiments or computational analyses neither does the FDA provide such services. Our external association operates on a finer scale involved also in the process that leads to the outcome.

## 3. Connection Between Quality Standards and Reproducible Research

A problem that is tightly connected to ensuring quality standards of data analysis results is reproducible research (Jasny et al., 2011). In recent years, it has been recognized that in times of increasing usage of advanced technologies for the generation of data, requiring also more sophisticated data analysis methods, ensuring the reproducibility of such studies is far from being trivial. In fact, many studies have been identified that are lacking this important requirement (Ioannidis et al., 2009; Nekrutenko and Taylor, 2012). For this reason, minimal standards have been established that should be followed (Sandve et al., 2013) and important elements of such standards include:

- documentation of all steps (data generation and data analysis).
- archive analysis software (including version control).
- store seed of random number generator.
- provide access to the analysis pipeline.

As a simple test for the reproducibility of a study it is often informative to ask a colleague from a different department to repeat the analysis, as described in the documentation. This may reveal problems with different versions of software (e.g., R packages), gaps in the documentation or inconsistencies in the preprocessing of the data. Hence, even such a simple test can be very helpful in spotting problems.

It is obvious that problems with the reproducibility of studies could be easily avoided by the involvement of an external association because general quality standards of a data analysis include the requirement of its reproducibility. This underlines that a wider look to a problem can be very beneficial because it can lead to the resolution/avoidance of related problems.

## 4. Practical Implementation

**Figure 1:**
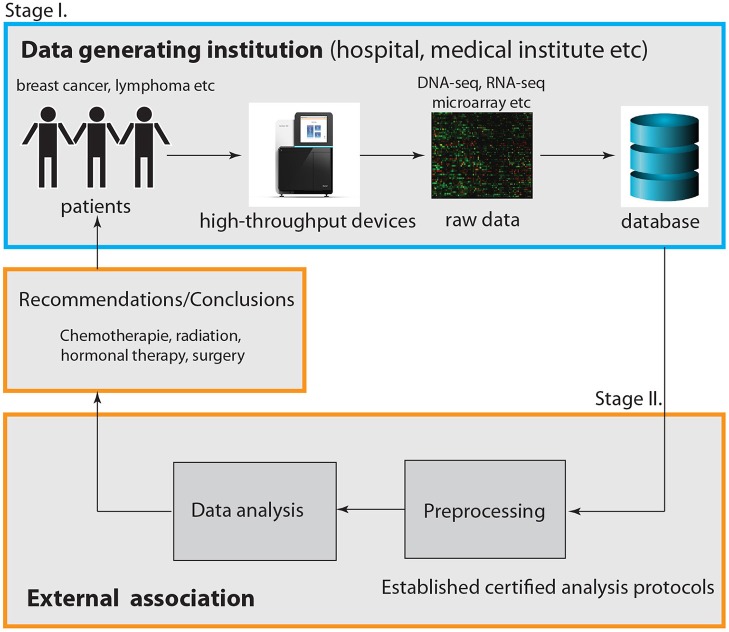
Visualization of the interplay between a data generating institution, e.g., a hospital or a medical institution, and an external association. Overall, this represents a two-stage cooperative service model.

So far we discussed the problem from a principle point of view. Now we turn to the practical implementation. In Figure 1, we summarize our discussion by providing a graphical visualization of the interplay between a data generating institution (outlined in blue) and an external association (outlined in orange). Here we distinguished visually between two components that are both part of the external association. The first involves all practical aspects of the data analysis including preprocessing and data integration, whereas the second one is only concerned with conclusions and recommendations derived from such an analysis.

Given the fact that many data generating institutions have already facilities for the analysis of data, e.g., biostatistics units, it would be efficient to utilize these in the following way. Instead of leaving it to the facilities to decide how to analyze the data, the external association should establish certified analysis protocols to follow. That means instead of performing the data analysis externally, it could be performed internally by the data generating institution itself, however, by following strict guidelines. In this way the lack of a research budget of facilities to establish optimal analysis protocols is compensated by the expertise of the external association.

As a side-effect, this would also deal with the problem of reproducibility because this is part of overall sound quality standards. Hence, only the part of the data analysis concerned with conclusions and recommendations should be under the sole governance of the external association.

## 5. Higher Standards by a Cooperation Service Model

We would like to emphasize that we consider the interplay between a data generating institution and an external association as a *cooperation*. The reason is that in research areas involving patients, the well-being and interests of the patients are top priority. This implies that it is not possible to keep analysis protocols shut way. In turn this means it will always be possible to reveal potential shortcomings or errors, as accomplished by Baggerly and Coombes (2009) and (Potti et al., 2011), because if undiscovered such errors will otherwise lead to physical or psychological harm of patients. Hence, in order to achieve the best possible outcome for the patients a cooperation between all involved parties is required that share responsibilities.

## 6. Quality Standards and Reproducible Research Beyond Oncology

The above discussion centered around cancer genomics data and the Duke Saga which happened in oncology (involving breast, colon, ovarian and lung cancer). However, we are of the opinion that such a collaborative model between a data generating institution and an external association as outlined above should be also beneficial for fields other than oncology.

A general characteristics of precision medicine and personalized medicine is that advanced data generation technologies are utilized in combination with sophisticated data analysis methods (Auffray et al., 2009; Ginsburg and Willard, 2009; Emmert-Streib and Dehmer, 2018). Since this is very similar to cancer genomics a corresponding translation of our outlined model should be transferable to other disease domains of precision medicine, e.g., immunology, neurodegenerative diseases or diabetes.

A current example that adds to our argument is given by Zolgensma. Zolgensma is an FDA approved gene therapy by Novartis intended to treat children with spinal muscular atrophy (SMA). This is the most severe form of SMA. On June 28 2019 the FDA was informed by AveXis about a data manipulation issue during product testing (FDA, 2019; Tirrell, 2019). This is also an example demonstrating the severity of the problem beyond oncology raised in this paper that can effect the life of patients and even children. Another example is the ban of the European Union of around 700 generic medicines for alleged manipulation of clinical trials conducted by the company GVK Biosciences (EMA, 2015).

## 7. Discussion

Due to the difficulty of the raised problem, we would like to clarify some further issues. First, our arguments are limited to biomedical studies involving either directly or indirectly patients. However, our arguments do not extend to general biological studies. The reason for this is that here we are not concerned with general reproducibility problems of studies but with consequences of such issues for patient treatment and care. That means, in our opinion, we focus on the most severe problem we are currently facing. If our arguments should also be extended to biological studies is open for discussion.

The crucial point is whether the outcome of an analysis from either diagnostic, prognostic or predictive investigations is affecting the treatment of patients with a clear causal connection between both. That means the analysis needs to inform the treatment. Hence, for instance an analysis of patient-derived cell lines, which only indirectly involves patients, falls under this category if the outcome of this analysis has a direct influence on the patient's treatment. It is clear that much of biomedical research is not directly concluding on any issue related to patient treatment, but instead typically investigates biological mechanisms.

Second, it is clear that the translation of biological findings toward their clinical usability is a long and difficult endeavor. As an example, we would like to mention the known difficulties of finding reproducible prognostic methylation biomarkers for colorectal cancer (Draht et al., 2018) or general cancers (Koch et al., 2018). This just underlines the difficulties we are facing experimentally and computationally requiring stringent protocols to safeguard against spurious results.

Third, in order to make an impact, we belief it is necessary to specify our scope precisely. The problem is that precision medicine or personalized medicine could refer to highly variable settings, ranging from basic research employing patient samples to decipher disease biology to drug development or clinical assessment. Unfortunately, these examples show that defining a scope precisely is not straight forward since all of these sub-studies relate to “*patients”*. For this reason, we suggest an assessment of the outcome of a study if it can potentially “*harm a patient.”* In this way the problem is converted to a legal issue and its definition is given by country-specific laws.

Fourth, data privacy is a current issue of great relevance in the big biomedical data era (Malin et al., 2013; Patil and Seshadri, 2014). However, our focus is on preventing patient harm due to inadequate data analysis standards. Of course, patients could also suffer from data privacy violations by third parties, however, not due to inadequate data analysis standards. Hence, data privacy is an issue data analysis services need to adhere to but for different reasons.

Fifth, erroneous data analysis results could come from deliberate cheating or other forms of scientific flaws. Interestingly, from a patient perspective, the potential harm is the same. However, safeguarding against the former threat is naturally accomplished by an external association because many of the incentives are eliminated in this way.

Sixth, problems of the sort discussed in this paper are actually rather widespread. For instance, in a survey study conducted in Bozzo et al. (2017) the authors identified 571 retracted publications in the cancer research literature of which 28.4% of the retrations were due to fraud and 24.2% due to errors. Further examples are provided in George and Buyse (2015) where the fabrication or falsification of data in clinical trials has been investigated effecting hundreds of publications in the literature.

## 8. Conclusions

A necessary step toward the practical realization for the certification of medical and clinical data analysis services would require the authorities to become active. For instance, legal laws could be legislated making the acquisition of such certificates mandatory attesting the fulfillment of quality standards. In turn, this would enable the establishment of external associations, similar to the model of the TÜV. If the Duke Saga could have been prevented is speculation. However, given the fact that Baggerly and Coombes (2009) were capable of reverse engineering some errors it appears reasonable to assume that an external association could have picked up these at an early stage before entering clinical trials.

In MacArthur (2012) it is argued that “Flawed papers cause harm beyond their authors: they trigger futile projects, stalling the careers of graduate students and postdocs, and they degrade the reputation of genomic research.” Importantly, we would like to add that flawed medical data analysis services can even severely harm the life of patients. In summary, we argued in favor of a “trust, but verify” approach because since the Duke Saga no legislative changes were initiated allowing history to repeat.

## Author Contributions

All authors contributed to all aspects of the preparation and the writing of the manuscript.

### Conflict of Interest

The authors declare that the research was conducted in the absence of any commercial or financial relationships that could be construed as a potential conflict of interest.
